# Aβ_8-20_ Fragment as an Anti-Fibrillogenic and Neuroprotective Agent: Advancing toward Efficient Alzheimer’s Disease Treatment

**DOI:** 10.1021/acschemneuro.2c00720

**Published:** 2023-03-01

**Authors:** Stefania Zimbone, Maria Laura Giuffrida, Giuseppina Sabatino, Giuseppe Di Natale, Rita Tosto, Grazia M. L. Consoli, Danilo Milardi, Giuseppe Pappalardo, Michele F.M. Sciacca

**Affiliations:** † Consiglio Nazionale delle Ricerche, Istituto di Cristallografia, Via Paolo Gaifami, 18, Catania 95126, Italy; ‡ Consiglio Nazionale delle Ricerche, Istituto di Chimica Biomolecolare, Via Paolo Gaifami, 18, Catania 95126, Italy

**Keywords:** Alzheimer, anti-aggregation, fibrils, oligomers

## Abstract

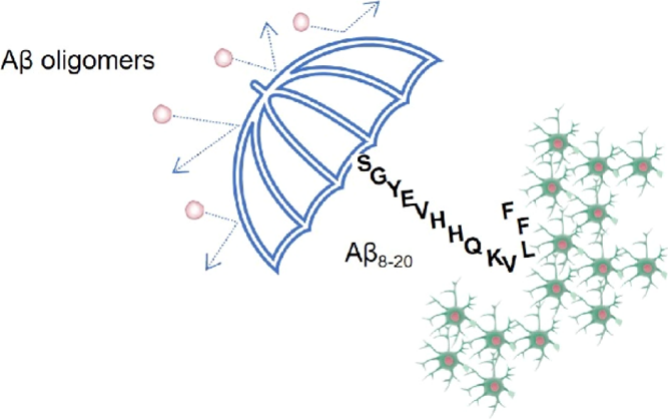

Alzheimer’s disease (AD) is the most common cause of dementia, characterized by a spectrum of symptoms associated with memory loss and cognitive decline with deleterious consequences in everyday life. The lack of specific drugs for the treatment and/or prevention of this pathology makes AD an ever-increasing economic and social emergency. Oligomeric species of amyloid-beta (Aβ) are recognized as the primary cause responsible for synaptic dysfunction and neuronal degeneration, playing a crucial role in the onset of the pathology. Several studies have been focusing on the use of small molecules and peptides targeting oligomeric species to prevent Aβ aggregation and toxicity. Among them, peptide fragments derived from the primary sequence of Aβ have also been used to exploit any eventual recognition abilities toward the full-length Aβ parent peptide. Here, we test the Aβ_8-20_ fragment which contains the self-recognizing Lys-Leu-Val-Phe-Phe sequence and lacks Arg 5 and Asp 7 and the main part of the C-terminus, key points involved in the aggregation pathway and stabilization of the fibrillary structure of Aβ. In particular, by combining chemical and biological techniques, we show that Aβ_8-20_ does not undergo random coil to β sheet conformational transition, does not form amyloid fibrils by itself, and is not toxic for neuronal cells. Moreover, we demonstrate that Aβ_8-20_ mainly interacts with the 4–11 region of Aβ_1-42_ and inhibits the formation of toxic oligomeric species and Aβ fibrils. Finally, our data show that Aβ_8-20_ protects neuron-like cells from Aβ_1-42_ oligomer toxicity. We propose Aβ_8-20_ as a promising drug candidate for the treatment of AD.

## Introduction

Alzheimer’s disease (AD) is considered the most common form of dementia in the elderly population.^1^ Dementia is a significant contributor to loss of independence, disability, and care home placement and represents one of the costliest long-term pathologies to society, with 85% of costs related to family or social care.^2^ According to data from the World Alzheimer Report, over 46.8 million people were affected by dementia worldwide in 2015, with a prevision of a doubling of this number in the next 20 years.^2−4^ To date, despite the high interest of the scientific community, the etiopathogenesis of the disease is not fully understood, and no drugs are still available for the treatment despite the large number of clinical trials^5^ and the intense research activity on synthetic and/or natural compounds.^6−11^

AD is characterized by the appearance in the hippocampal region of the brain^12^ of two kinds of proteinaceous deposits: (i) one mainly constituted by fibrillar aggregates of phosphorylated tau protein inside neuronal cells, called neurofibrillary tangles,^13^ and (ii) one in the extracellular space, called amyloid plaques, mainly constituted by fibrillar aggregates of amyloid beta protein (Aβ).^14^ Amyloid plaque composition was demonstrated to be a complex mixture with the presence of 100s of proteins (∼500) and several non-proteinaceous components.^15−17^

Aβ is the final product of the cleavage of amyloid precursor protein (APP)^18^ operated by the sequential action of β and γ secretases. Although Aβs spanning 34 to 50 amino acid length are the most common, there are even shorter Aβ isoforms (Aβ1–17/18/19/20) that depend on γ-secretase, but the precise mechanism of their generation is unknown.^19−21^ It was proposed that an abnormally high concentration of Aβ_1-40_ and/or Aβ_1-42_, the two most common isoforms, could result in aggregation into a β-sheet-rich structure, the starting point of Aβ fibrillogenesis.^22,23^ Aβ aggregation is a complex mechanism which starts with the formation of oligomeric species, suggested to be the most toxic species for cells,^24−31^ which undergo conformational reorganization into protofibrils and fibrils. Monomeric and fibrillar forms have been demonstrated to be, respectively, protective^32−34^ and mostly inert^35,36^ for neuronal cells.

Many studies focused on the primary sequence of Aβ, trying to shed light on the role played by a single amino acid.^37^ Although a high amount of data is available, the literature is often contradictory. It has been shown that the mainly hydrophobic C-terminal region of Aβ plays a pivotal role in controlling Aβ structure stability and self-assembly.^38−41^ On the contrary, it is generally accepted that the N-terminal region of Aβ, Aβ_1-16_, is not able to aggregate and is not cytotoxic.^42^ Nevertheless, it was also shown that under particular conditions, Aβ_1-16_ can aggregate and form cytotoxic species containing β-turns.^43^ Moreover, the N-terminal region of Aβ was demonstrated to control the aggregation rate and fibrillar stability of amyloid fibers.^44^ In particular, residues Arg5, Asp7, and Ser8 were found to form important inter-molecular contacts stabilizing the overall fibril structure of three-fold symmetry.^45^

Aβ undergoes several post-translational modifications,^19^ including the formation of truncated species as the result of physiological enzymatic cleavage.^46−49^ Many truncated forms of Aβ have been identified in blood plasma samples and human cerebrospinal fluids of AD patients.^50^ Recently, Abedin et al. reported a structural and aggregation propensity study of seven Aβ fragments with the aim of identifying the region of Aβ which is able to inhibit fibrillogenesis.^51^ Generally, truncated forms of Aβ are of particular interest since they are known to affect the Aβ aggregation rate. Short Aβ fragments known as β-sheet breakers are able to recognize and tie to the same regions of the parent amyloid peptide, effectively inhibiting accumulation or promoting disaggregation of pre-existing fibrillar amyloids.^52−58^ In particular, β-sheet breakers Aβ_17-21_ and Aβ_16-20_ (KLVFF) have been shown to significantly inhibit amyloidogenic aggregation in vitro.^59−63^ Unfortunately, these peptide-based systems have a remarkable tendency to self-aggregate and short circulatory half-lives. For this reason, the inclusion of charged residues at the N-terminus could be thought of as a valuable strategy to enhance bioavailability. Here, we explore the ability of the Aβ fragment SGYEVHHQKLVFF (Aβ_8-20_) to prevent the aggregation and toxicity of Aβ_1-40_ and Aβ_1-42_. The choice of this peptide, which has not yet been found in vivo, arises from two main reasons:

(i) the absence of Arg5 and Asp7 is important to prevent the stabilization of the fibril structure.^45^ Moreover, it is known that angiotensin-converting enzyme is a candidate enzyme for the formation of the 8-*x* Aβ species,^64^ although so far, there are no in vivo data supporting this pathway;
(ii) the cleavage in position 20 removes a major part of the Aβ C-terminus region which is known to play an important role in the aggregation process. Moreover, it encompasses the Lys-Leu-Val-Phe-Phe (KLVFF) sequence which was demonstrated to recognize the analogous sequence in the full-length protein.^65^ Indeed, several studies were performed on KLVFF alone or embedded in the peptide sequence,^6,52,66,67^ indicating the ability of the KLVFF sequence to both recognize and prevent Aβ aggregation.

We used chemical and biochemical techniques to fully evaluate the behavior and the properties of Aβ_8-20_. We show that Aβ_8-20_ invariably maintains a random coil conformation and is not able to form amyloid aggregates by itself. Interestingly, Aβ_8-20_ suppresses the Aβ_1-40_ and Aβ_1-42_ random coil to β-sheet conformational transition and completely prevents their ability to form amyloid aggregates. We also demonstrate, through a combination of mass spectrometry and dot blot assay, that this peptide hampers the formation of Aβ_1-42_ oligomeric species, which are considered the most toxic species, probably by interacting in the 4–11 region of the protein. Finally, we show that Aβ_8-20_, which is not toxic *per sé*, protects neuronal-like cells from Aβ_1-42_ toxicity. Overall, our data indicate Aβ_8-20_ as a good candidate for the prevention of cell damage induced by Aβ in AD and help us improve our knowledge of the mechanism underlying the detrimental action of oligomeric and/or prefibrillar Aβ species.

## Results and Discussion

### Aβ_8-20_ Adopts a Stable Random Coil Conformation and Does Not Form Amyloid Aggregates

**Figure 1:**
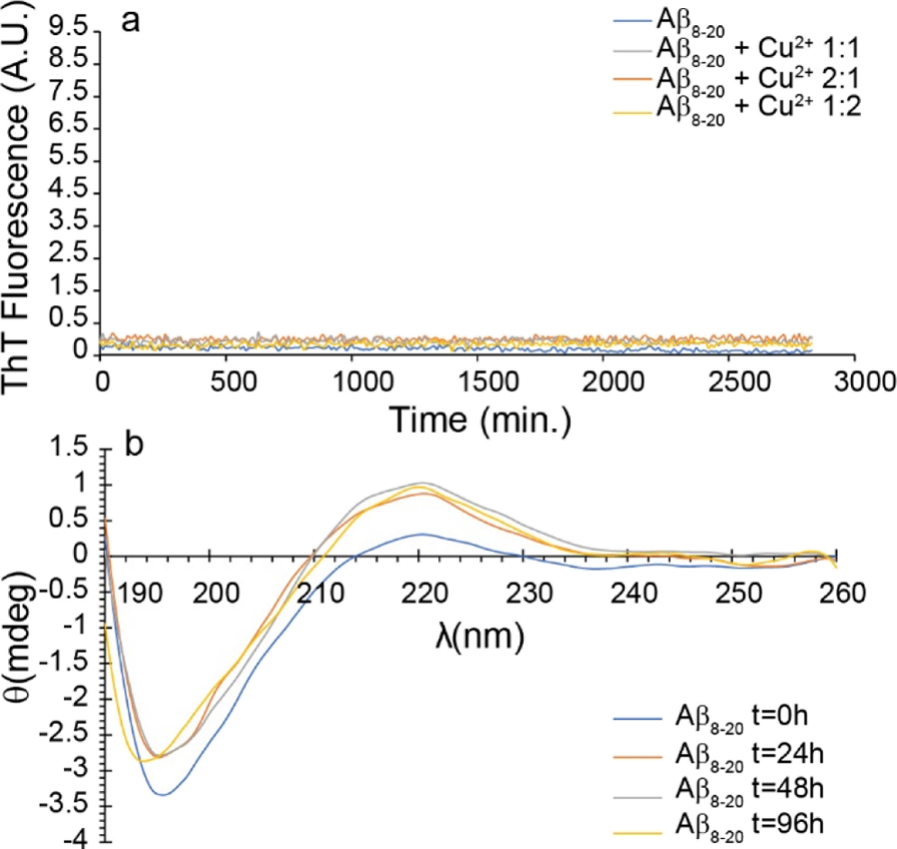
(a) Amyloid aggregation measured by the ThT assay of 10 μM Aβ_8-20_ (blue curve), Aβ_8-20_:Cu^2+^ 1:1 (gray curve), Aβ_8-20_:Cu^2+^ 2:1 (orange curve), and Aβ_8-20_:Cu^2+^ 1:2 (yellow curve). (b) Secondary structure measured by CD of 10 μM Aβ_8-20_ at *t* = 0 (blue curve), *t* = 24 h (orange curve), *t* = 48 h (gray curve), and *t* = 96 h (yellow curve). All the experiments were performed in 10 mM MOPS buffer and 100 mM NaCl, pH 7.4. ThT curves are the average of three independent experiments.

Several fragments of Aβ have been shown to undergo amyloidogenic aggregation.^51,68^ Although Aβ_8-20_ lacks the aggregation-prone C-terminus region and residues in the N-terminus responsible for amyloid fiber stabilization, we could not rule out the possibility that this fragment may form amyloid aggregates. To evaluate the aggregation properties of Aβ_8-20_, we initially performed a well-known thioflavin T (ThT) assay. Aβ_8-20_ in buffer solution (10 mM MOPS buffer, 100 mM NaCl, pH 7.4) does not aggregate over a time length of 48 h (Figure 1a, blue curve).

To further test the amyloidogenic properties of the peptide, we performed ThT experiments also under more complex fibrillogenic conditions. Interestingly, also the presence of the Cu^2+^ ion, which is well known to strongly modulate the aggregation of Aβ depending on the ion/protein ratio,^69−73^ in sub-stoichiometric (Figure 1a, orange curve), stoichiometric (Figure 1a, gray curve), and over-stoichiometric (Figure 1a, yellow curve) ratios does not induce any aggregation of Aβ_8-20_.

Circular dichroism (CD) experiments, performed in a time range of 96 h (Figure 1b), reveal that Aβ_8-20_ adopts a random coil conformation over time. Interestingly, our results differ from those obtained by Abedin et al. for the Aβ_11-20_ fragment which shows, despite the high sequence homology, a high propensity to form a β-sheet-rich structure.^51^ The absence of any significant secondary structure transition, typical of amyloid fiber formation, supports well ThT results, although it is not possible to exclude the formation of amorphous aggregates. However, the stable intensity of ellipticity measured by CD experiments suggests that Aβ_8-20_ does not form any insoluble aggregates over time.

### _Aβ8-20_ Prevents Aggregation and Random Coil to β-Sheet Transition of Both Aβ_1-40_ and Aβ_1-42_

**Figure 2:**
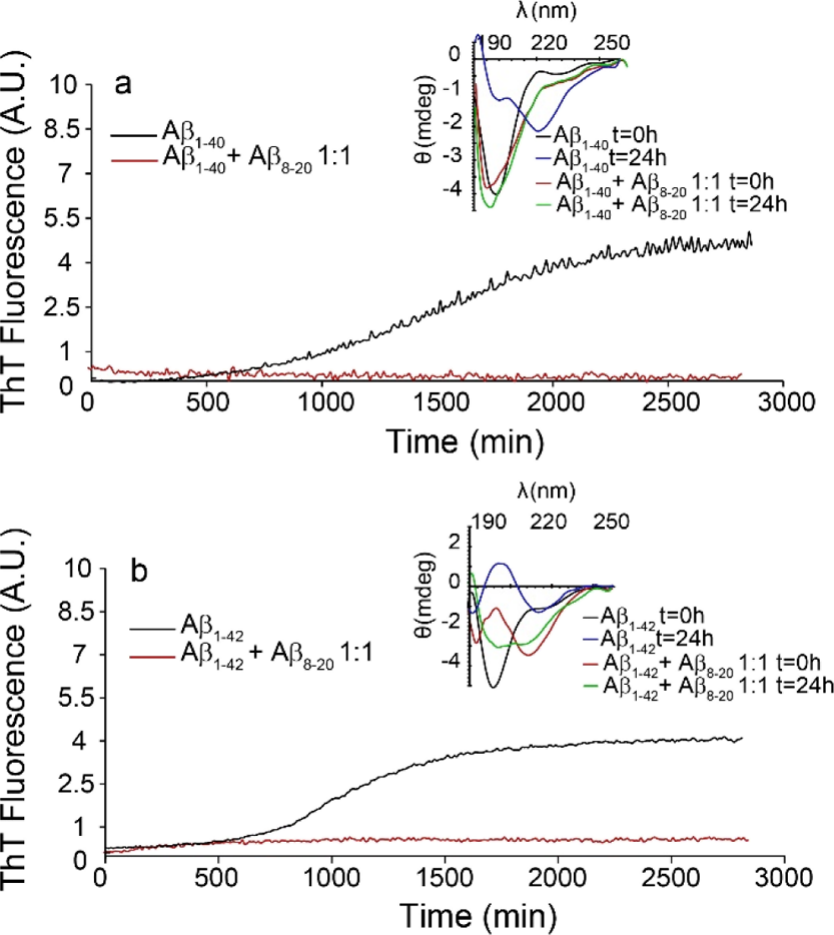
(a) Amyloid aggregation measured by ThT assay of 10 μM Aβ_1-40_ (black curve) and Aβ_1-40_:Aβ_8-20_ 1:1 (red curve). Inset: CD spectra of 10 μM Aβ_1-40_ at *t* = 0 (black curve) and *t* = 24 h (blue curve) and Aβ_1-40_:Aβ_8-20_ 1:1 at *t* = 0 (red curve) and *t* = 24 h (green curve). (b) Amyloid aggregation measured by ThT assay of 10 μM Aβ_1-42_ (black curve) and Aβ_1-42_:Aβ_8-20_ 1:1 (red curve). Inset: CD spectra of 10 μM Aβ_1-42_ at *t* = 0 (black curve) and *t* = 24 h (blue curve) and Aβ_1-42_:Aβ_8-20_ 1:1 at *t* = 0 (red curve) and *t* = 24 h (green curve). All the experiments were performed in 10 mM MOPS buffer and 100 mM NaCl, pH 7.4. ThT curves are the average of three independent experiments, and the single traces and error bar are reported in the Supporting Information (Figure S3).

Aggregation of both Aβ_1-40_ and Aβ_1-42_ peptides in buffer solution shows the typical sigmoidal ThT curve (Figure 2a black curve and Figure 2b black curve). CD spectra show, over time, a random coil to β-sheet secondary structure transition (Figure 2a inset and Figure 2b inset). Aβ_1-42_, which is considered the most toxic species,^74,75^ forms amyloid fibers faster than Aβ_1-40_, which, in turn, is the most abundant species in vivo^76−79^ but is considered less toxic. Noteworthy, the presence of Aβ_8-20_ in a 1:1 concentration ratio completely suppresses Aβ_1-40_ aggregation (Figure 2a red curve) and prevents the random coil to β-sheet transition (Figure 2a inset) after 24 h incubation. A similar effect was observed for samples containing Aβ_1-42_ (Figure 2b, black curve). Aβ_1-42_ in the presence of Aβ_8-20_ shows only a small residual increase in the ThT signal (Figure 2b, red curve). Interestingly, CD spectra (Figure 2b, inset) reveal a mixture of the random coil and β-sheet structure at *t* = 0 which evolves over 24 h into a random coil/α-helix. Thus, our data suggest that Aβ_8-20_ could interfere with the aggregation process of both Aβ_1-40_ and Aβ_1-42_ already at a 1:1 molar ratio, suggesting a higher efficiency than, for example, that of Aβ_11-20_.^51^

**Figure 3:**
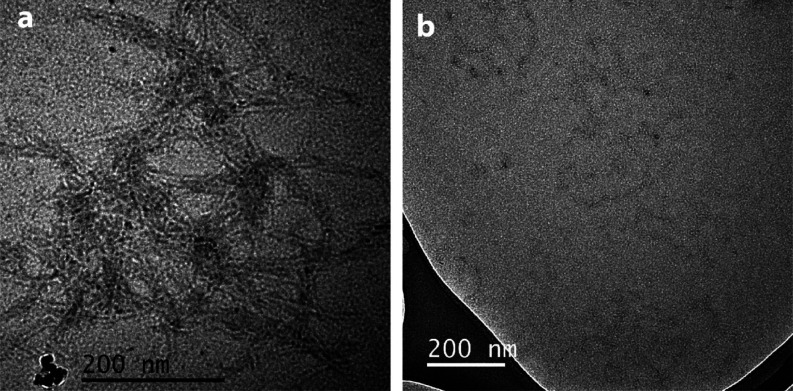
TEM image of (a) Aβ_1-42_ 100 μM after 96 h incubation and (b) Aβ_1-42_ 100 μM: Aβ8-20 100 μM after 96 h incubation.

To confirm ThT results, we acquired transmission electron microscopy (TEM) images for samples containing Aβ_1-42_ 100 μM alone (Figure 3a) and Aβ_1-42_ 100 μM: Aβ_8-20_ 100 μM (Figure 3b) after 96 h incubation at 37 °C. β_1-42_ showed the classical fiber network, while the presence of the Aβ_8-20_ fragment almost completely inhibited the fiber formation of Aβ_1-42_ which were not detected over the entire surface of the grid. This result confirms what we observed by ThT experiments.

### Aβ_8-20_ Reduces the Dimension of Aβ_1-40_ Soluble Aggregates

**Figure 4:**
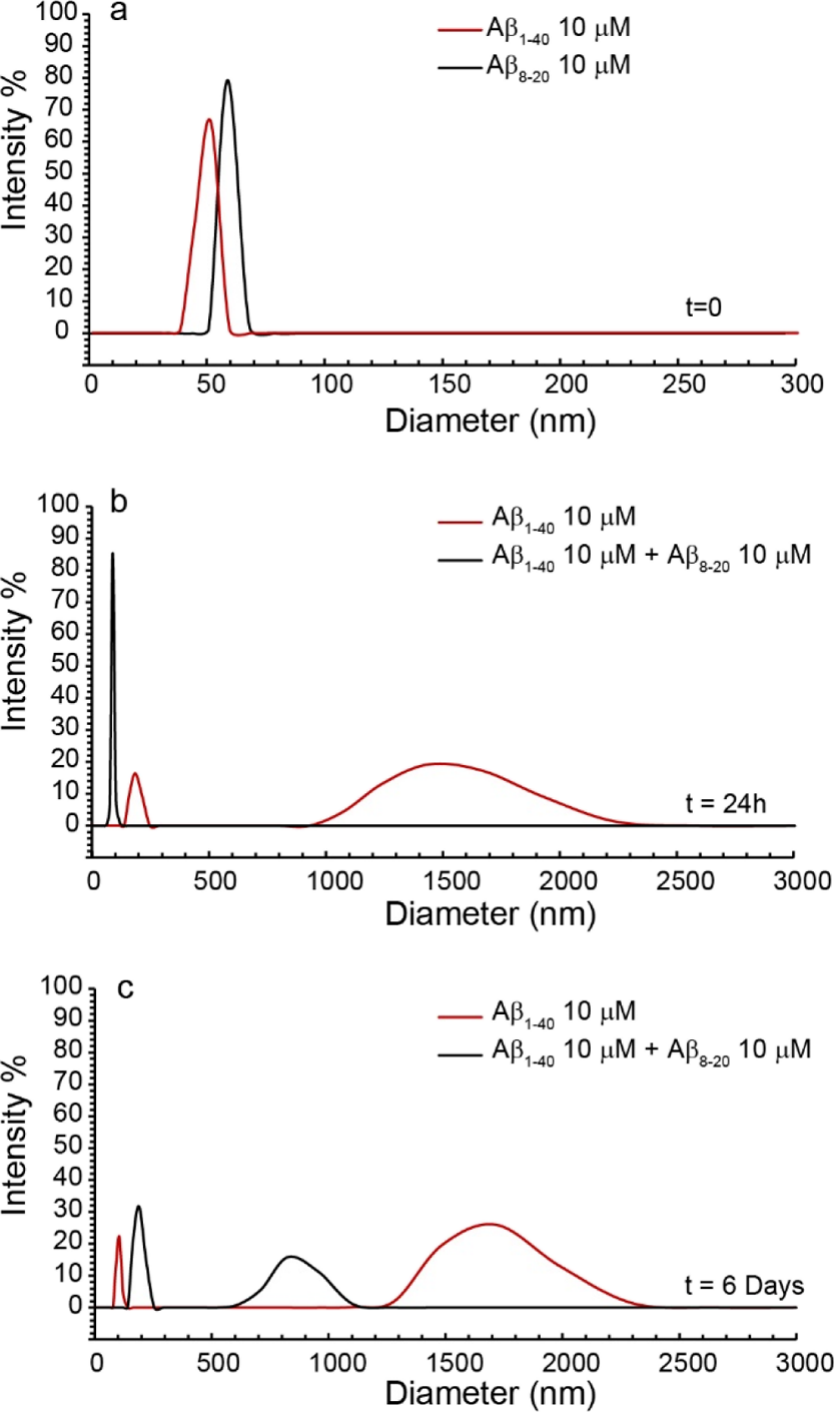
Intensity-weighted hydrodynamic diameter distribution for (a) 10 μM Aβ_1-40_ and Aβ_8-20_ at *t* = 0 and (b) 10 μM Aβ_1-40_ (red line) and Aβ_1-40_:Aβ_8-20_ 1:1 concentration ratio (black line) at *t* = 24 h and (c) at *t* = 6 days. Curves are the average of three independent experiments. Measures were performed at 37 °C in 10 mM phosphate buffer.

To evaluate the effect of the presence of Aβ_8-20_ on the size distribution of soluble species of Aβ, we resorted to dynamic light scattering (DLS) measurements. We chose the Aβ_1-40_ isoform since it is known to aggregate more slowly than Aβ_1-42_, giving us enough time to evaluate the dimension of soluble aggregated species. Thus, the growth of the Aβ_1-40_ aggregates was monitored in the absence and in the presence of Aβ_8-20_. Data collected at *t* = 0, 24 h, and 6 days as the analysis of the intensity (%) of scattering objects are reported in Figure 4. The analysis indicated that freshly prepared samples of Aβ_1-40_ and Aβ_8-20_ form structures with mean hydrodynamic diameter around 50 and 60 nm, respectively (Figure 4a, red and black curves, respectively). After 24 h, in the sample containing only Aβ_1-40_, aggregation phenomena generated a population of larger aggregates with size centered at 1505 nm (64%) in addition to a population centered at 190.9 nm (36%), whereas only a population centered at 116 nm was observed in the presence of Aβ_8-20_ (Figure 4b). After 6 days, two main populations with mean hydrodynamic diameter centered at 1663 (60%) and 102.7 nm (40%) and at 850.3 (33%) and 188.7 nm (67%) were observed for Aβ_1-40_ alone and in the presence of Aβ_8-20_, respectively (Figure 4c). The data collected clearly indicated the reduction of the dimension of Aβ_1-40_ soluble aggregates in the presence of the Aβ_8-20_ fragment.

### _Aβ8-20_ Hampers Aβ_1-42_ Oligomer Formation

Since Aβ_1-42_ oligomers have been demonstrated to be the main species responsible for Aβ toxicity in vitro and in vivo,^24−27^ we resorted to matrix-assisted laser desorption ionization mass spectrometry (MALDI-MS) to investigate the effect of the Aβ_8-20_ peptide on the formation of Aβ_1-42_ oligomers. Indeed, MALDI-MS can acquire the *m*/*z* values of peptides and proteins predominately in the singly charge state, enabling a direct indication of the mass of Aβ_1-42_ oligomers. In particular, the *m*/*z* values reported in the mass spectra acquired by MALDI-MS give a direct indication of the mass of peptides and proteins, revealing the monomeric/multimeric composition of Aβ samples.^80−82^ Nevertheless, a drawback of the MALDI approach in the characterization of multimeric forms of Aβ_1-42_ was the use of organic solvent acetonitrile (ACN) during sample preparation. In particular, the ACN/H_2_O (1:1, *v/v*) solvent mixture, needed for both matrix dissolution and rapid evaporation of the solvent after the deposition of the sample on target plates (see the Materials and Methods section), prevents the hydrophobic interactions within oligomers affecting the oligomer composition.^83^ Therefore, we carried out MALDI experiments at Aβ_1-42_ concentrations higher (100 μM) than those generally used in MALDI investigations (5–10 μM) to aid the formation of high-molecular weight (MW) oligomers^84^ and prevent the complete dissolution of oligomers during deposition of the sample (Aβ/matrix mixture) on target plates.

**Figure 5:**
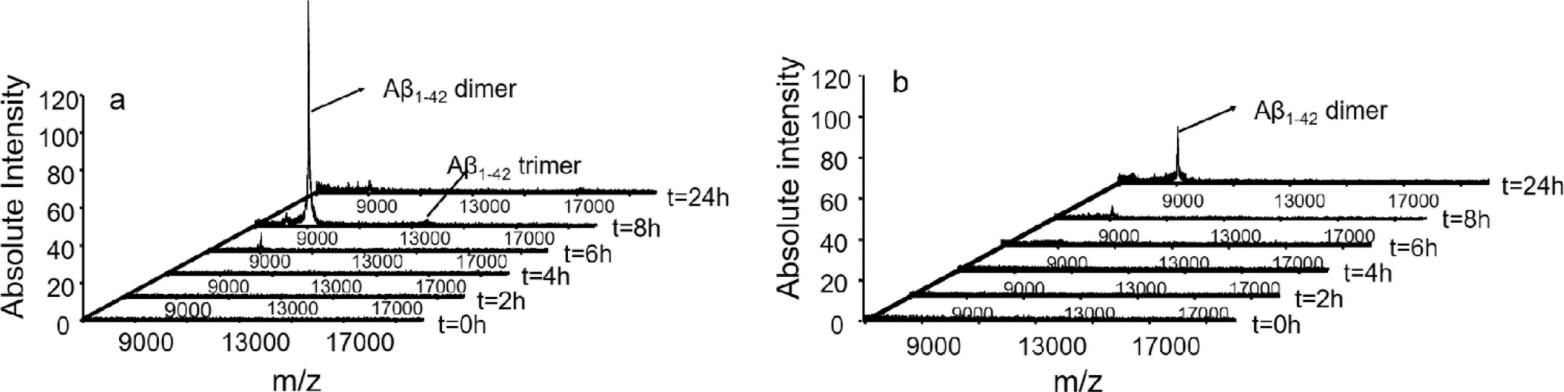
MALDI-MS spectra acquired in the linear mode (*m*/*z* range = 7000–20,000) at different incubation times of (a) Aβ_1-42_ (*c* = 100 μM) in PBS buffer (5 mM) pH 7.8 and (b) Aβ_1-42_/Aβ_8-20_ in PBS buffer (5 mM) pH = 7.8 (*c*_Aβ_ = *c*_Aβ8-20_ = 100 μM).

An Aβ_1-42_ sample was analyzed by MALDI-time of flight (TOF) after 2, 4, 6, 8, and 24 h of incubation at 37 °C. The mass spectra acquired (Figure 5a) were compared with those recorded when an equimolar amount of the Aβ_8-20_ peptide was added to the Aβ_1-42_ sample solution (Figure 5b).

The MALDI-TOF spectrum recorded after 8 h of incubation showed (Figure 5a) the formation of a series of signals corresponding to dimeric {[(Aβ_1-42_)_2_ + *H*]^+^*m*/*z* = 9029} and trimeric {[(Aβ_1-42_)_3_ + *H*]^+^*m*/*z* = 13540} oligomers (Figure 5a). These signals could be related to the formation of high-MW oligomers that were partially disrupted when the Aβ_1-42_ sample was mixed with matrix solution. Interestingly, the mass spectrum of the sample containing the Aβ_1-42_/Aβ_8-20_ mixture, recorded after 8 h of incubation (Figure 5b), showed a clear reduction of the signal’s intensity corresponding to the Aβ_1-42_ oligomers. The *m*/*z* signal corresponding to the Aβ_1-42_ dimer can be observed only in the mass spectrum acquired after 24 h of incubation.

These findings are in keeping with the results observed in ThT experiments and support the hypothesis that Aβ_8-20_ may interfere with Aβ aggregation by means of the formation of a noncovalent adduct with the amyloid peptide.

**Figure 6:**
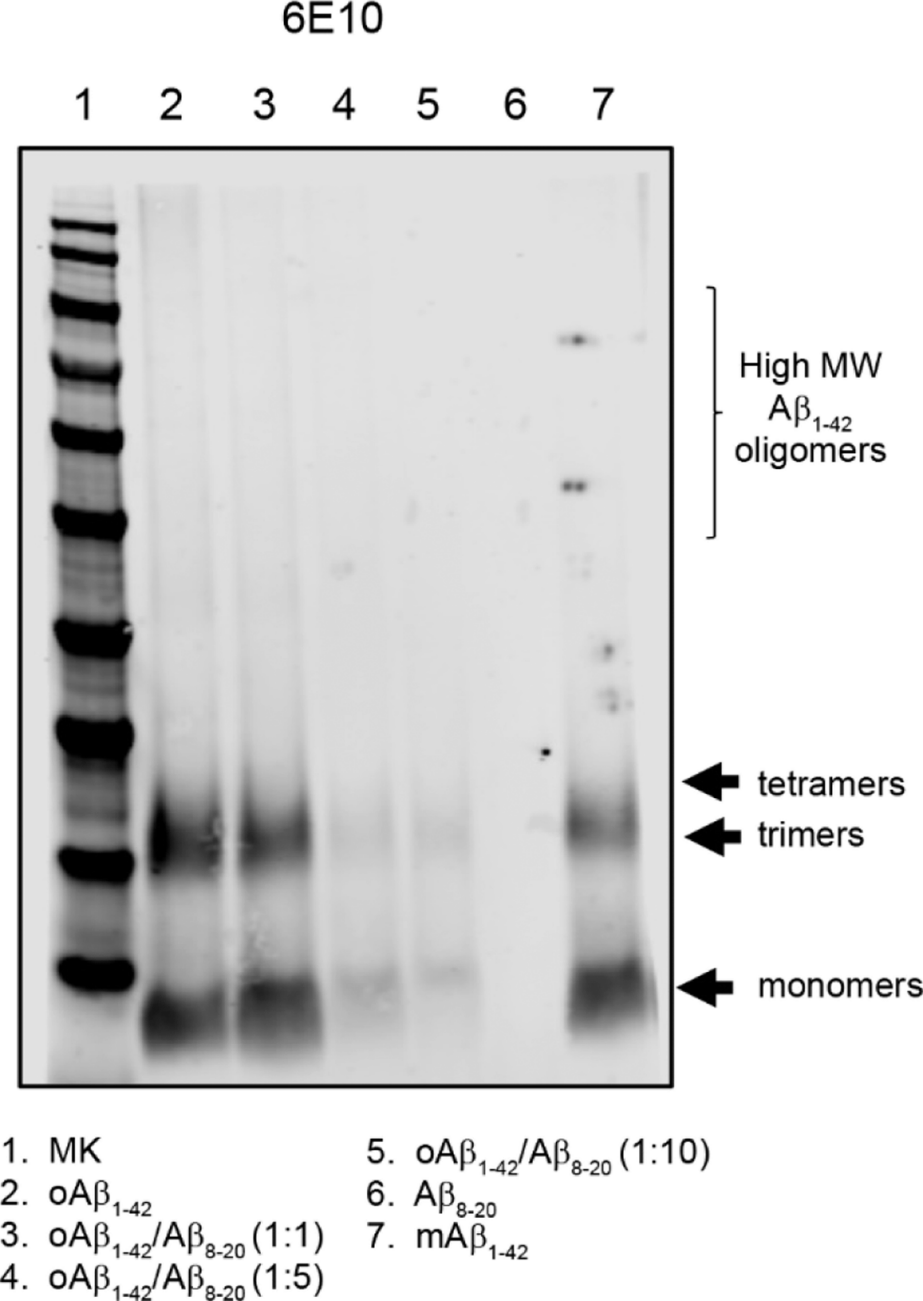
Representative western blot of Aβ oligomers prepared in the presence or absence of Aβ_8-20_. Samples were separated onto a 4–12% bis·tris SDS-PAGE gel and blotted with anti-Aβ N-terminal 1–16 mouse monoclonal antibody 6E10 (1:500).

To further confirm the interaction between Aβ_8-20_ and Aβ_1-42_ and investigate oligomer formation, we performed gel electrophoresis after incubation of freshly prepared Aβ_1-42_ in the presence or in the absence of Aβ_8-20_ for 48 h at 4 °C. We tested three different molar ratios of Aβ_1-42_/Aβ_8-20_ (1:1; 1:5; and 1:10), and after incubation, each sample was characterized for its composition of Aβ aggregates. The samples were loaded onto a polyacrylamide gel and transferred onto a nitrocellulose membrane (Figure 6). As expected, we found that Aβ_1-42_ alone aggregates into small oligomers ranging from 8 to 16 kDa, representing dimers, trimers, and tetramers. In the presence of Aβ_8-20_, the signal observed was less intense when incubated at the molar ratio of 1:1 and became very weak at 1:5 and 1:10. These data suggest the ability of Aβ_8-20_ to bind the full-length peptide, inhibiting its aberrant aggregation by hampering oligomer formation.

### _Aβ8-20_ Interacts with the N-Terminal Region of Aβ_1-42_

**Figure 7:**
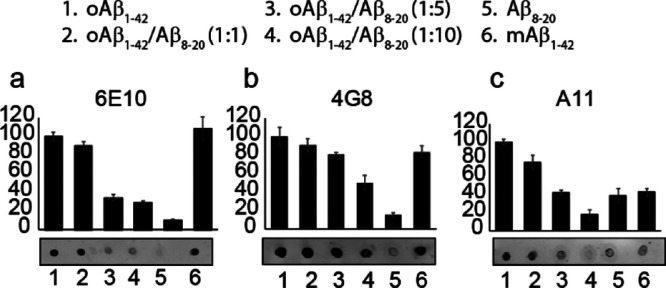
Dot blot analysis of Aβ oligomers prepared in the presence or absence of Aβ_8-20_. Samples were spotted after 48 h incubation at 4 °C under gentle rotation. Membranes were blotted with the following antibodies: anti-Aβ N-terminal 1–16 mouse monoclonal antibody 6E10 (1:100); anti-Aβ 17–24 mouse monoclonal antibody 4G8 (1:100); or anti-oligomer A11 rabbit polyclonal antibody (1:100).

Noteworthily, it is clear from Figure 6 that samples containing Aβ_1-42_/Aβ_8-20_ in molar ratios 1:5 and 1:10 not only show a decrease in the intensity of trimer and tetramer signals, but also monomer bands seem to disappear. To better investigate this unexpected result, we spotted the co-incubated samples Aβ_1-42_/Aβ_8-20_ at the three different molar ratios (1:1; 1:5; 1:10) onto a nitrocellulose membrane that we probed with the 6E10 antibody (Figure 7a). We used Aβ_1-42_ oligomers and freshly prepared monomers as controls. Even in this case, we found that when Aβ_8-20_ was incubated with Aβ_1-42_ at more than the 1:1 molar ratio, a clear signal decrease was evident. Controls confirmed that the antibody was working properly, revealing the presence of Aβ in both incubated and freshly spotted samples.

As for electrophoresis data, these results suggest that the presence of the peptide strongly modulates Aβ_1-42_ self-assembly. To prove this, we used a different antibody, anti-Aβ 4G8 (Figure 7b), which is reported to react to amino acid residues 17–24 with the epitope lying within amino acids 18–22 of β-amyloid (VFFAE). Targeting a different epitope led to a different signal pattern in which we detected a clear staining even in the case of co-incubated samples, revealing that the lack of 6E10 signals previously observed could be due to the presence of the small peptide Aβ_8-20_ along the Aβ_1-42_ reactive sequence.

**Scheme 1:**
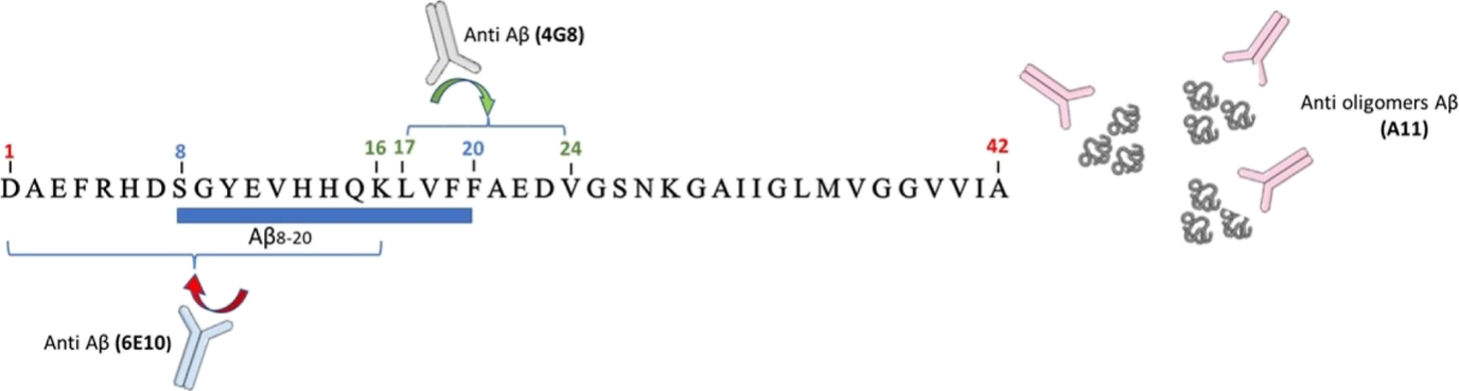
Schematic Representation of Anti-Aβ Antibody Interaction with Aβ_1-42_ in the Presence of the Aβ_8-20_ Fragment

6E10 is, in fact, directed against amino acids 1–16 of the Aβ sequence. During the incubation time, the binding of Aβ_8-20_ to Aβ_1-42_ could hinder the interaction between the antibody and its target sequence (Scheme 1).

We finally used the anti-oligomer antibody A11 to assess the conformation of each spotted sample and the ability of Aβ_8-20_ to effectively interfere with Aβ_1-42_ assembly. As expected, A11 strongly reacted with the Aβ incubated alone and very slightly with Aβ monomers freshly spotted. The increasing presence of the Aβ_8-20_ fragment during the incubation time leads to a decrease in the antibody signal underlying the reduction of the oligomeric species formed.

On the basis of these findings, we moved onto limited proteolysis experiments to better clarify the site of interaction between Aβ_8-20_ and Aβ_1-42_. Indeed, these interactions could occur at the peptide bonds involved in the proteolytic cleavage affecting, in turn, enzyme’s accessibility to the cleavage sites.

**Scheme 2:**
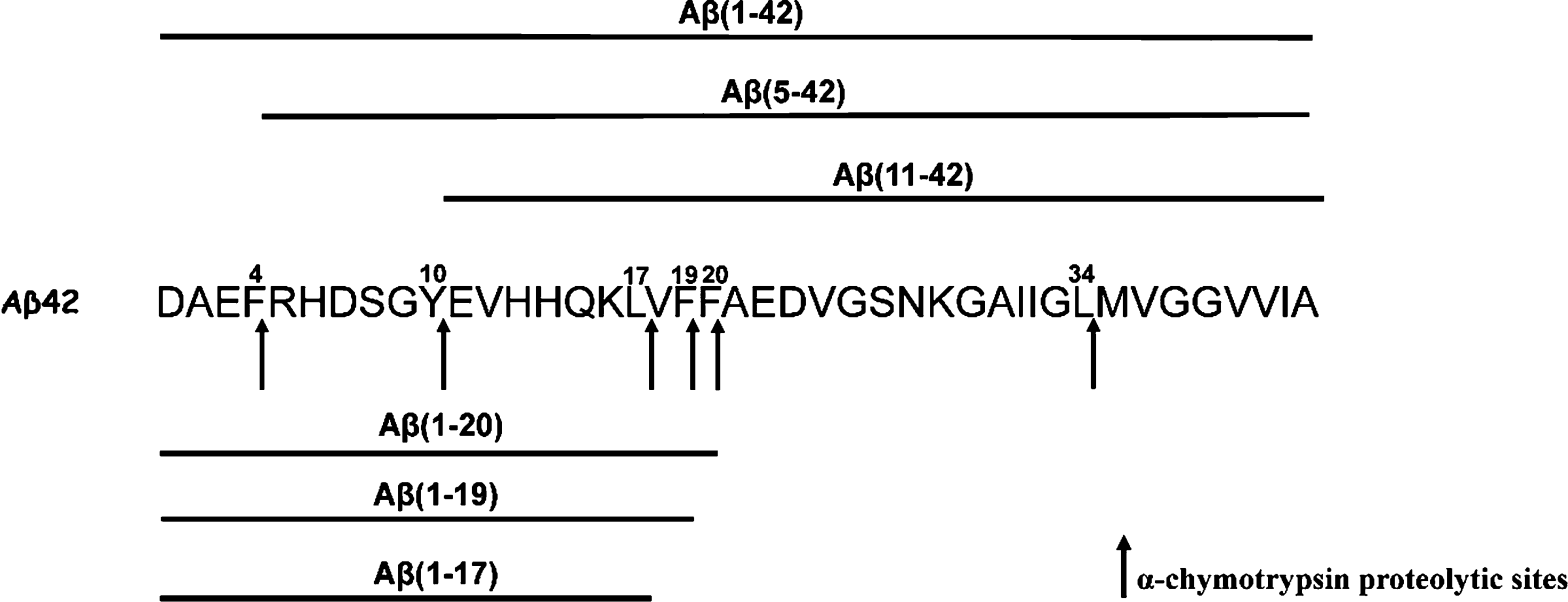
Aβ_1-42_ Proteolytic Pattern after 10 min of α-Chymotrypsin Digestion Aβ_1-42_ (10 μM) in PBS buffer (5 mM) pH 7.8 and an enzyme/substrate ratio of 1:200 w/w.

To this scope, we used α-chymotrypsin enzyme that selectively catalyzes the hydrolysis of peptide bonds at the C-terminal side of tyrosine, phenylalanine, tryptophan, and leucine residues. We analyzed by MALDI-TOF the Aβ_1-42_ peptide fragments generated at the initial stage, namely, after 10 min of α-chymotrypsin digestion, where the hydrolysis rate is higher, and small differences in the peptide interactions with Aβ_1-42_ would be more pronounced, as observed in our previous studies.^6^ The identified peptide fragments are indicated in Scheme 2. A quite similar proteolytic pattern was observed in the MALDI-TOF spectrum of Aβ_1-42_ digested in the presence of the Aβ_8-20_ peptide.

**Figure 8:**
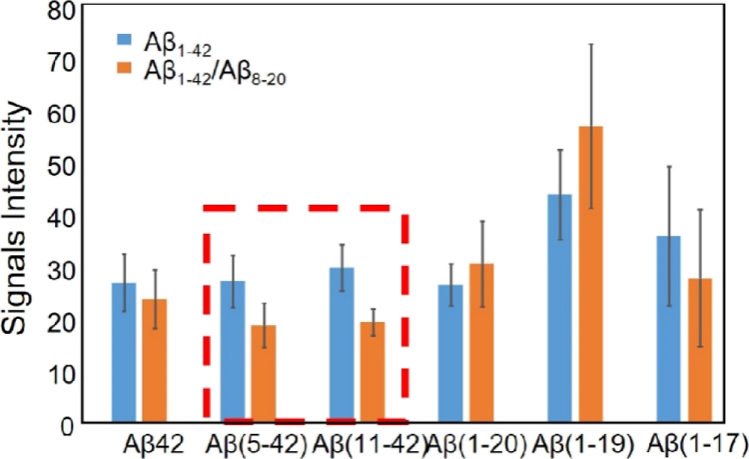
Signal intensities of digestion fragments of Aβ_1-42_ protein in Aβ_1-42_ (blue bar) and Aβ_1-42_/Aβ_8-20_ samples (orange bar), after 10 min of α-chymotrypsin digestion.

Despite the low reproducibility of MALDI measurements, a comparative analysis of the signal intensity averaged over 15 replicate measurements (Figure 8) revealed some reasonable differences. In particular, the reduction of signal intensity of the peaks assigned to the peptide fragments Aβ_5-42_ and Aβ_11-42_, when the Aβ_8-20_ peptide was added to the Aβ_1-42_ sample solution (Figure 8, orange bar), indicates a lower hydrolysis rate at the cleavage sites of Phe4 and Tyr10. This may suggest a lower accessibility of these cleavage sites. Interestingly, the CD spectra of Aβ_1-42_ in the presence of Aβ_8-20_ show a mixture of the random coil and β-sheet structure at *t* = 0 h (Figure 2b inset, red curve) which evolves over 24 h into a random coil/α-helix conformation (Figure 2b inset, orange curve). The structuring effects within the polypeptide backbone can alter peptide chain flexibility of the Aβ_1-42_ N-terminal domain, affecting the cleavage of the peptide bonds by a protease.

### Aβ_8-20_ Prevents Aβ_1-42_ Toxicity in Differentiated SH-SY5Y Cells

**Figure 9:**
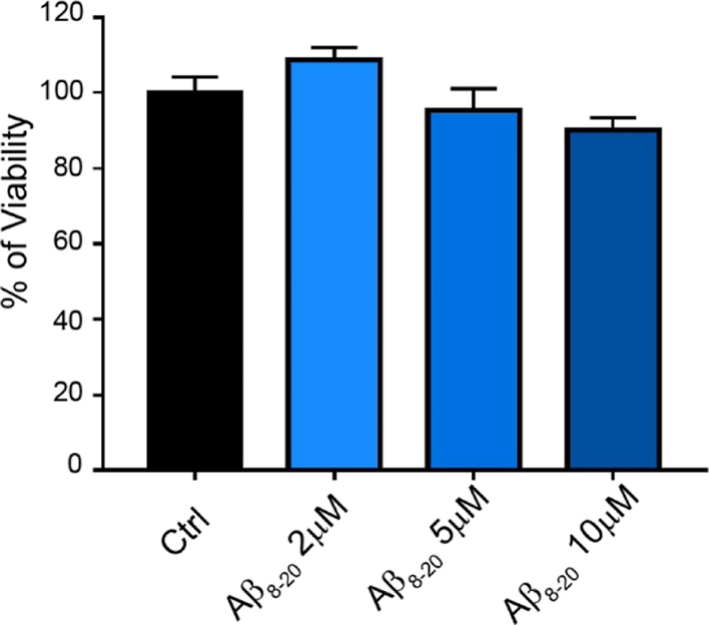
MTT assay of fully differentiated SH-SY5Y cells treated for 48 h with increasing concentrations of Aβ_8-20_ (2, 5, and 10 μM). Bars represent means ± SEM of three independent experiments with *n* = 3 each.

To have a functional readout of the data, we investigated the protective activity of Aβ_8-20_ both *per sé* and toward the toxicity of Aβ_1-42_ oligomers by using the well-known viability test, 3-(4 5-dimethylthiazol-2-yl)-2,5-diphenyltetrazolium bromide (MTT) assay. We used a neuronal-like model obtained by the neuroblastoma cell line, SH-SY5Y, fully differentiated with all-trans-retinoic acid (RA). Aβ_8-20_ did not show any toxic activity. Even at higher concentrations and after 48 h exposure, Aβ_8-20_ was not significantly toxic for the cell, whose viability was comparable to that of controls (Figure 9).

**Figure 10:**
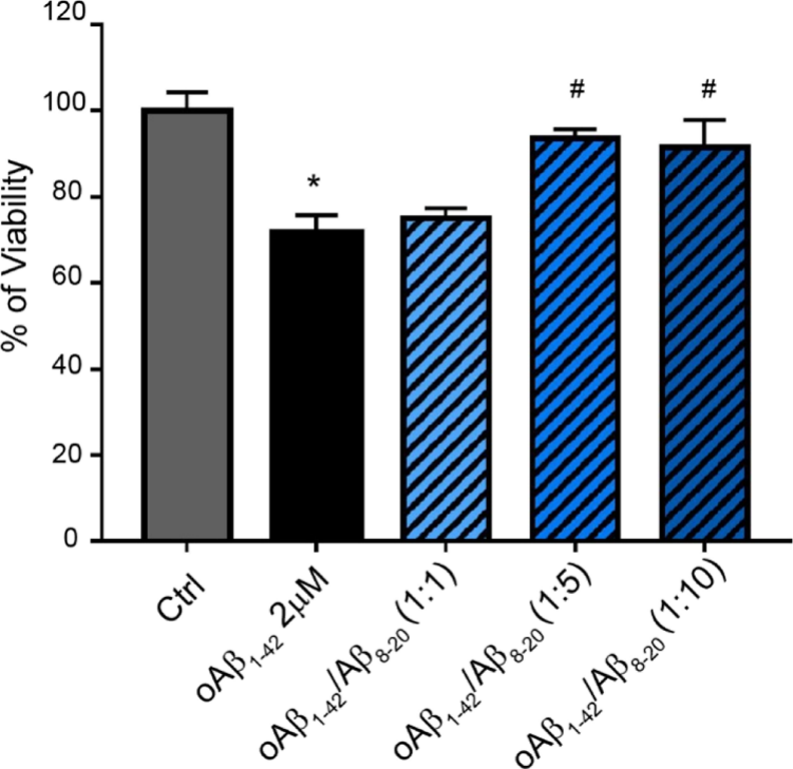
MTT assay of fully differentiated SH-SY5Y cells treated for 48 h with Aβ oligomers prepared in the presence or absence of different molar ratios of Aβ_8-20_ (1:1; 1:5; and 1:10). Samples were incubated at 4 °C under gentle rotation for 48 h. Bars represent means ± SEM of three independent experiments with *n* = 3 each. ***P < 0.001 vs Ctrl by one-way ANOVA + Tukey test and # <0.001 vs Aβ_1-42_ by one-way ANOVA + Tukey test.

The ability of Aβ_8-20_ to prevent oligomer toxicity was tested by incubating Aβ_1-42_ for 48 h at 4 °C alone or in combination with Aβ_8-20_, added at the molar ratios of 1:1; 1:5; and 1:10, previously used for dot blot analysis. After incubation, cells were exposed to oligomers at the final concentration of 2 μM, and the resulting toxicity was compared to the effects of the other co-incubated solutions (Figure 10). As expected, after 48 h of treatments, oligomers were toxic, affecting the cell viability by approximately a 30% reduction. Unlike the lower (2 μM) concentration, both 1:5 and 1:10, corresponding, respectively, to 10 and 20 μM Aβ_8-20_, were able to counteract oligomer toxicity.

## Conclusions

Over the past years, the anti-aggregating properties of several natural compounds, synthetic derivatives, and peptides have attracted the attention of the scientific community as potential drugs for the treatment of AD. Furthermore, the growing evidence supporting a role for Aβ in neuronal physiology^32,34,85^ has highlighted the need to find novel potential drugs capable of blocking the progression of the disease and preserving the natural functions of Aβ, which are normally lost following its self-assembly. Although difficult due to their transient nature, oligomers represent the main targets to be addressed, while the activity of Aβ is still a matter of investigation and only partially clarified. To date, none of the proposed molecules have been successfully proven to halt and/or prevent the pathology.

In this paper, we present data about a newly synthesized fragment of Aβ, encompassing residues 8–20 (Aβ_8-20_), which has shown promising features to be considered in AD therapy.

Aβ_8-20_ freshly solubilized has a hydrodynamic diameter of ∼60 nm (Figure 4a) and assumes a random coil conformation which does not evolve into a β-sheet-rich structure over time (Figure 1b) according to the absence of any amyloid structure as evidenced by ThT assay under several conditions (Figure 1a). Thus, as expected, the lack of the hydrophobic C-terminus and residues Arg5 and Asp7 involved in fiber stabilization in the N-terminal region of the protein hampers the formation of the amyloid structure. Notably, this peptide is not toxic toward cells (Figure 9) up to a concentration of 10 μM. Aβ_8-20_ almost completely inhibits fiber formation of both Aβ_1-40_ and Aβ_1-42_ (Figure 2a,b, respectively, and Figure 3) which mostly remains in a random coil conformation over time (Figure 2a inset and Figure 2b inset, respectively). Moreover, the presence of Aβ_8-20_ significantly reduces the dimension of soluble aggregated species of Aβ_1-40_ over time (Figure 4b,c), suggesting a strong modulation of oligomer formation. This hypothesis was corroborated by MALDI experiments combined with dot blot analysis which clearly show that Aβ_8-20_ efficiently reduced the formation of the Aβ_1-42_ dimer and trimer species (Figure 5) in a concentration-dependent way (Figure 6). The presence of the self-recognizing KLVFF sequence suggests that Aβ_8-20_/Aβ_1-42_ interaction should occur in the N-terminal region as confirmed by the dot blot analysis (Figure 7) which shows that the interaction of Aβ_8-20_ with Aβ_1-42_ hinders the link between the 6E10 antibody and its target sequence (1–16) on Aβ. Limited proteolysis experiments confirm these data, indicating the 4–11 region as the most involved in the interaction (Figure 8). Finally, Aβ_8-20_ proved to completely protect, in a dose-dependent way, SH-SY5Y cells from the toxicity of Aβ_1-42_ oligomers (Figure 10).

The whole of our results clearly indicates Aβ_8-20_ as an interesting Aβ fragment that combines the required β-sheet breaker activity with promising features such as the lack of toxicity on neuronal cultures and the effective protective properties against oligomer-mediated cellular death.

These properties make the fragment a good candidate for a potential disease-modifying drug in AD therapy, encouraging a more in-depth study on the biological and molecular features of the peptide.

## Materials and Methods

### Reagents

Aβ 1–40 (Aβ_1-40_) and Aβ 1–42 (Aβ_1-42_) were purchased from Bachem (Bubendorf, Switzerland) with a purity of ≥95%. Amyloid fragments 8–20 (Aβ_8-20_) were synthesized and purified in our laboratory with a purity of ≥98%. ThT, ascorbic acid, NaCl, and all other salts were purchased from Sigma-Aldrich (St. Louis, MO, USA). The MALDI matrices 3,5-dimethoxy-4- hydroxycinnamic acid (sinapinic acid, SIN) and α-cyano-4-hydroxycinnamic acid (α-CHCA) were purchased from Sciex and used without further purification. Bovine serum albumin, immunoglobulin G, and peptide mass standard calibration kits were purchased from Sciex. ACN and trifluoroacetic acid (TFA) (mass spectrometry-grade) were purchased from Fisher Scientific. Alpha-chymotrypsin was purchased from Sigma-Aldrich. All aqueous solutions were prepared using a Barnstead NanoPure system with a 0.2 mm membrane filter (Thermo Scientific).

### Amyloid Fragment 8–20 Synthesis

The Aβ 8–20 (Aβ_8-20_) were synthesized by a fully automated microwave-assisted solid-phase peptide synthesis following the Fmoc/*t*Bu strategy on a liberty peptide synthesizer (CEM) starting from Rink amide AM resin (substitution 0.59 mmol/g). After the resin swelling in dimethylformamide (DMF), all orthogonally protected Fmoc amino acids were introduced according to the following N,N′-Diisopropylcarbodiimide (DIC)/Oxyma activation method consisting of (1) Fmoc deprotections (20% piperidine in DMF); (2) washes (3×) with DMF; (3) couplings with protected amino acids (5 equiv, 0.2 M in DMF), Oxyma pure (5 equiv, 1 M in DMF), and DIC (5 equiv, 0.5 M in DMF) prepared in separate bottles; and (4) washes (3×) with DMF. The following instrumental conditions were used for each coupling cycle: (a) 220 W, 65 °C, 30 s and (b) 25 W, 90 °C, 90 s. The instrumental conditions used for the deprotection cycle were (a) 220 W, 70 °C, 30 s and (b) 25 W, 75 °C, 30 s. After the last Fmoc deprotection, N-terminal acetylation was carried out with Ac_2_O (100 μL/200 mg of resin) in DMF (2 × 10 min). The cleavage of the peptide from the resin, with concomitant deprotection of acid-labile amino acid side-chains, was achieved by treatment of peptide–resin with TFA/Triisopropylhydrosilane/H_2_O (95:2.5:2.5 v/v/v, 10 mL) for 2.5 h at room temperature and with magnetic stirring. The resin was filtered, and the crude peptide was recovered by precipitation with freshly distilled diisopropyl ether. The purification of crude Aβ_8-20_ was carried out by preparative reversed-phase high-performance liquid chromatography (RP-HPLC) using a SHIMADZU LC-20A chromatography system equipped with an SPD-M20A photodiode array detector with detection at 222 and 254 nm. A Jupiter 10u Proteo C12 250 × 21.2 mm (90 Å pore size, AXIA Packed) column was used. The peptides were eluted at a flow rate of 10 mL/min according to the following protocol: from 0 to 3 min, isocratic conditions in 90% solvent A (H_2_O containing 0.1% TFA) followed by a 3 min linear gradient from 10 to 45% B (CH_3_CN containing 0.1% TFA) and then a 4 min linear gradient from 45 to 50% B and finally 5 min isocratic conditions in 50% B. Fractions containing the desired product were collected and lyophilized. The purity of the peptide was checked by analytical RP-HPLC using a Phenomenex Kinetex XB- C18 analytical column (pore size: 100 A, particle size: 5 μm, column length: 250 mm, and internal diameter: 4,60 mm). A linear gradient of ACN (containing 0.1% TFA) and water (containing 0.1% TFA) (90:10 water–acetonitrile to 0:100 water–acetonitrile over 15 min and at a flow rate of 1 mL min^–1^) was used. Sample identity was confirmed by MALDI-MS. Calculated mass 1632.81; observed: [M + H]^+^ = 1632.96; [M + Na]^+^ = 1653.87; [M + K]^+^ = 1670.85.

### Peptide Preparation

To prevent the presence of any preformed aggregates, Aβ_1-40_, Aβ_1-42_, and Aβ_8-20_ were initially dissolved in hexafluoroisopropanol (HFIP) at a concentration of 1 mg/mL and then lyophilized overnight. To be used for the experiments, the lyophilized powder was initially dissolved in 1 mM NaOH to obtain a stock solution with a final concentration of 100 μM. Each stock solution was used immediately after preparation by diluting it in the opportune buffer solution to reach the concentration needed for experiments.

### Thioflavin T Assay

Kinetics of Aβ_1-40_, Aβ_1-42_, and Aβ_8-20_ fiber formation was measured using ThT assay. Samples were prepared by diluting, in 3-(N-morpholino)propanesulfonic acid (MOPS) buffer, stock solution of Aβ_8-20_, Aβ_1-40_, Aβ_1-42_, or a combination to reach the final concentration. Copper, where present, was added from a stock solution at the indicated concentration. ThT was then added to a final concentration of 20 μM. Experiments were carried out in Corning 96-well non-binding surface plates. Time traces were recorded using a Varioskan (Thermo Fisher, Walham, MA) plate reader using a λ_ex_ of 440 nm and a λ_em_ of 485 nm at 37 °C, shaking the samples for 10 s before each read. All ThT curves represent the average of three independent experiments.

### Circular Dichroism

CD spectra were acquired using a J-810 spectrometer (Jasco, Japan) under a constant flow of N_2_ at room temperature. The CD spectra were recorded for Aβ_1-40_ and Aβ_1-42_ (10 μM) monomers in the absence and presence of Aβ_8-20_ (10 μM) in the 1:1 molar ratio. The lyophilized samples were dissolved in 1 mM NaOH and then diluted to obtain a concentration of 10 μM for Aβ alone and the mixture. The CD measurements were carried out in aqueous solution (1 × 10^–3^ M MOPS buffer and 0.05 M NaF). A 0.5 cm path length quartz cuvette was used to acquire the far-UV CD spectra (190–260 nm), at a scan speed of 50 nm/min. 10 scans were collected. The measurements were performed in triplicate. The CD intensities were expressed as θ(mdeg).

### Transmission Electron Microscopy

Samples were prepared by incubating at 37 °C for 96 h 100 μL of phosphate buffer solutions 10 mM and 100 mM NaCL, pH 7.4 containing Aβ_1-42_ 100 μM or Aβ_1-42_ 100 μM: Aβ_8-20_ 100 μM. After incubation, 3 μL of each sample was deposited onto a copper grid and allowed to adsorb for 5 min before the grid was rinsed with H_2_O twice. Samples were dried overnight and then stained with uranyl acetate. TEM micrographs were acquired using JEOL JEM 2010F using a 2 K × 2 K Gatan ORIUS camera. Samples have been observed in low magnification using the image formed inside the transmitted beam on the focal plane. This method is useful in low-magnification detection of weak objects, without any change in the focus configuration of the microscope. By switching between the out-of-focus image in the focal plane and the in-focus image in the image plane, it is possible to scan a large area of the grid with enhanced detection capability and easily come back to the normal image configuration.

### Dynamic Light Scattering

DLS measurements were carried out on a Zetasizer NanoZS90 Malvern Instrument (UK) equipped with a 633 nm laser at a scattering angle of 90° and at 37 °C. The samples of Aβ_1-40_ and Aβ_8-20_ (5 μM) were prepared under the same experimental conditions as those described above. Each measurement was performed three times.

### Mass Spectrometry

MALDI mass spectra were obtained using a 5800 MALDI-TOF/TOF mass spectrometer (Sciex) equipped with an automated single-plate sample-loading system, 1 kHz OptiBeam On-Axis Laser Nd/YAG 349 nm wavelength, delayed extraction (DE), two acceleration regions, a two-stage reflector mirror, and a 1000 MHz digitizer. The instrument was operated in the reflectron mode (m/range: 800–5000) and linear mid-molecular weight mode (m/range: 7000–20,000). When operating in the linear mode, the instrument’s acquisition parameters were set to optimize detection sensitivity of Aβ oligomers. In particular, the lowest possible laser intensity was used to minimize dissociation and enable the detection of Aβ_1-42_ oligomers. Moreover, the detection of high-MW oligomers was hindered by the presence of the monomers. Therefore, mass spectra were acquired starting at higher *m*/*z* values to enhance the sensitivity for the large-MW species. DE was applied, and the delay time was set according to the MW of the analytes to optimize resolution of their molecular ion. Mass spectra were acquired by averaging 300 to 600 shots. Sinapinic acid and α-CHCA were prepared by dissolving 10 mg of matrices in 1 mL of 50% ACNe in 0.05% TFA and 1 mL of 30% ACN in 0.1% TFA, respectively. Standard kits were used to calibrate the mass scale of the MALDI mass spectrometer. The peptide mass standard kit includes des-Arg1-Bradikynin, angiotensin I, Glu1-Fibrinopeptide B, adrenocorticotropic hormone (ACTH) (clip 1–17), ACTH (clip 18–39), and ACTH (clip 7–38), and it was used to cover a mass range of 800 to 4000 Da. Bovine insulin, *E. coli* thioredoxin, and horse apomyoglobin were used to cover a mass range from 4000 to 20,000 Da. Aβ_1-42_ samples were monomerized to remove any preformed aggregates using the procedure described above. Stock 1 solutions of Aβ_1-42_ and Aβ_8-20_ were prepared by dissolving 0.1 mg of each lyophilized peptide in HFIP (stock 1 = 1.5 mM). An opportune amount of each stock solution was diluted in phosphate buffer solution (5 mM, pH 7.8) to a concentration of 50 μM (dtock 2) and mixed to obtain stock solutions to be used for the experiments. The Aβ_1-42_ sample and the equimolar mixtures of Aβ_1-42_/Aβ_8-20_ were prepared from stock 2 solutions and stock 1 solution for a final concentration of 5 μM for limited proteolysis experiments and 100 μM for oligomer experiments, respectively. For limited proteolysis experiments, a fresh stock of α-chymotrypsin (1.0 mg/mL) was made with HCl (1 × 10^–3^ mol dm^–3^), and then, an appropriate volume of the enzyme stock solution was added to Aβ_1-42_ and Aβ_1-42_/Aβ_8-20_ samples for a final enzyme/substrate ratio of 1:200 w/w. Solutions were incubated at 25 °C for 10 min. For MALDI-TOF measurements, samples were analyzed using the dried-droplet preparation methods. In particular, 1 to 2 μL of the sample and 1 to 2 μL of matrix solution were mixed into a 0.5 mL tube, and 1 μL of this mixture was deposited on a stainless steel 384-well plate. The mixture samples on the plate were dried by evaporation of the solvent at room temperature till a thin microcrystalline layer of the sample/matrix occurred. All the samples were spotted in three different wells of the plate (triplicate), and five mass spectra were recorded for every spot. MS data were imported into freely available open-source software mMass (http://www.mmass.org). Mass spectra acquired for each sample (15 spectra) were averaged, and monoisotopic peaks were automatically picked. Theoretical *m*/*z* values of Aβ_1-42_, Aβ_8-20_, and peptides resulting from in silico digestion of amyloid protein were compared with the *m*/*z* values assigned to experimental mass spectra. Peptides matching successfully, within a tolerance of 0.05 Da, were annotated. Moreover, mass spectra were exported as a peak list and processed using Excel (Microsoft) software to evaluate the 95% confidence interval of each signal intensity assigned.

### Dot Blot Analysis

The Aβ_1-42_ sample (100 μM) was incubated for 48 h at 4 °C under gentle rotation in the presence or absence of different molar ratios of Aβ_8-20_ (1:1; 1:5; and 1:10). Then, samples were spotted onto a nitrocellulose membrane. The membrane was blocked with Odyssey blocking buffer (LiCor, Biosciences) at room temperature for 1 h. After blocking, the membrane was probed overnight at 4 °C and with gentle shaking with the following antibodies: anti-Aβ N-terminal 1–16 mouse monoclonal antibody 6E10 (1:100) (BioLegend), anti-Aβ 17–24 mouse monoclonal antibody 4G8 (1:100) (BioLegend), or anti-oligomer A11 rabbit polyclonal antibody (1:100) (Invitrogen, Thermo Fisher). Finally, the membrane was repeatedly washed and exposed to the anti-mouse antibody labeled with the IRDye secondary antibody (1:20.000 Li-Cor Biosciences) for 45 min at room temperature. Hybridization signals were detected with the Odyssey CLx Infrared Imaging System (LI-COR Biosciences).

### Western Blot Analysis

Aβ_1-42_ (100 μM) alone and in combination with different molar ratios of Aβ_8-20_ (1:1; 1:5; and 1:10) was incubated at 4 °C for 48 h to form Aβ oligomers. After incubation, the amount and size of Aβ aggregates were determined by Western blot analysis. A volume of 25 μL of each unheated sample was loaded onto a precast Bis-Tris gel (Bolt 4–12%, Life Technologies) with 2-morpholin-4-yl ethanesulfonic acid. Samples were transferred onto a nitrocellulose membrane (0.2 mm, Hybond ECL, Amersham Italia) by using a wet transfer unit Mini Blot Module (Life Technologies). Membranes were blocked in Odyssey blocking buffer (Li-COR Biosciences) and incubated at 4 °C overnight with anti-Aβ N-terminal 1–16 mouse monoclonal antibody 6E10 (1:500) (BioLegend). A secondary goat anti-mouse antibody labeled with infrared dye (1:20.000) was used at room temperature for 45 min. Hybridization signals were detected with the Odyssey CLx infrared imaging system (LI-COR Biosciences, Lincoln, NE).

### Cell Culture and MTT Assay

The neuroblastoma cell line, SH-SY5Y, was maintained in Dulbecco’s modified Eagle’s medium (DMEM)-F12 (Gibco, Thermo Fisher) supplemented with 10% heat-inactivated (HI) fetal calf serum (Gibco, Thermo Fisher), 100 mg/mL penicillin and streptomycin (Gibco, Thermo Fisher), and 2 mM l-glutamine at 37 °C and 5% CO_2_. Two weeks before experiments, 5 × 10^3^ cells were plated on 96-well plates in DMEM-F12 with 5% HI fetal calf serum. The percentage of serum was gradually decreased until it was 1% of the total. All-trans-RA (Sigma), 5 μM, was used to promote neuronal differentiation, and medium-containing RA was changed every 3 days. Fully differentiated SH-SY5Y cells were treated with increasing concentrations of Aβ_8-20_ (2, 5, and 10 μM). After 48 h treatment, cultures were incubated with MTT (5 mg/mL) for 2 h at 37 °C and then lysed with dimethyl sulfoxide (DMSO), and the formazan production was evaluated in a plate reader through the absorbance at 570 nm.

### Anti-Oligomerization Activity

To prepare Aβ_1-42_ oligomers, 1 mg of Aβ_1-42_ (HFIP-treated) was first dissolved in 5 mM DMSO. A solution of 100 μM Aβ_1-42_ in ice-cold DMEM F-12 without phenol red was prepared and allowed to oligomerize for 48 h at 4 °C according to the Lambert protocol^86^ with some modifications as previously described.^32^ To evaluate the ability of Aβ_8-20_ to inhibit toxic Aβ oligomerization, Aβ_1-42_ was incubated in the presence or absence of different molar ratios of Aβ_8-20_ (1:1; 1:5; and 1:10). After 48 h incubation at 4 °C under gentle rotation, Aβ_1-42_/Aβ_8-20_ samples were applied to the differentiated SH-SY5Y cells at the final concentration of 2 μM Aβ_1-42_. The ability of Aβ_8-20_ to prevent Aβ oligomer formation and toxicity was evaluated by measuring cell viability after 48 h treatment by MTT assay.

## Supporting Information Available

Chromatographic profile of Aβ_8-20_; FT-MS spectra of Aβ_8-20_; and single ThT traces of Aβ_1-40_ and Aβ_1-42._ (PDF)
