# Supplementary material for: Aβ8-20 Fragment as an Anti-Fibrillogenic and Neuroprotective Agent: Advancing toward Efficient Alzheimer’s Disease Treatment
Source: ACS Chem Neurosci. 2023 Mar 1;14(6):1126–36. doi: 10.1021/acschemneuro.2c00720 (PMC10020970; doi:10.1021/acschemneuro.2c00720)
Supplement: Supplementary file 1 — cn2c00720_si_001.pdf [file cn2c00720_si_001.pdf]

# Supporting information

# The Aβ8-20 fragment as anti-fibrillogenic and neuroprotective agent: advancing towards efficient Alzheimer's disease treatment

Stefania Zimbone†, Maria Laura Giuffrida†, Giuseppina Sabatino†, Giuseppe Di Natale†, Rita Tosto†, Grazia M. L. Consoli‡, Danilo Milardi†, Giuseppe Pappalardo†* and Michele F.M. Sciacca†*

†Consiglio Nazionale delle Ricerche, Istituto di Cristallografia, Via Paolo Gaifami, 18, Catania, Italy.

‡Consiglio Nazionale delle Ricerche, Istituto di Chimica Biomolecolare, Via Paolo Gaifami, 18, Catania, Italy.

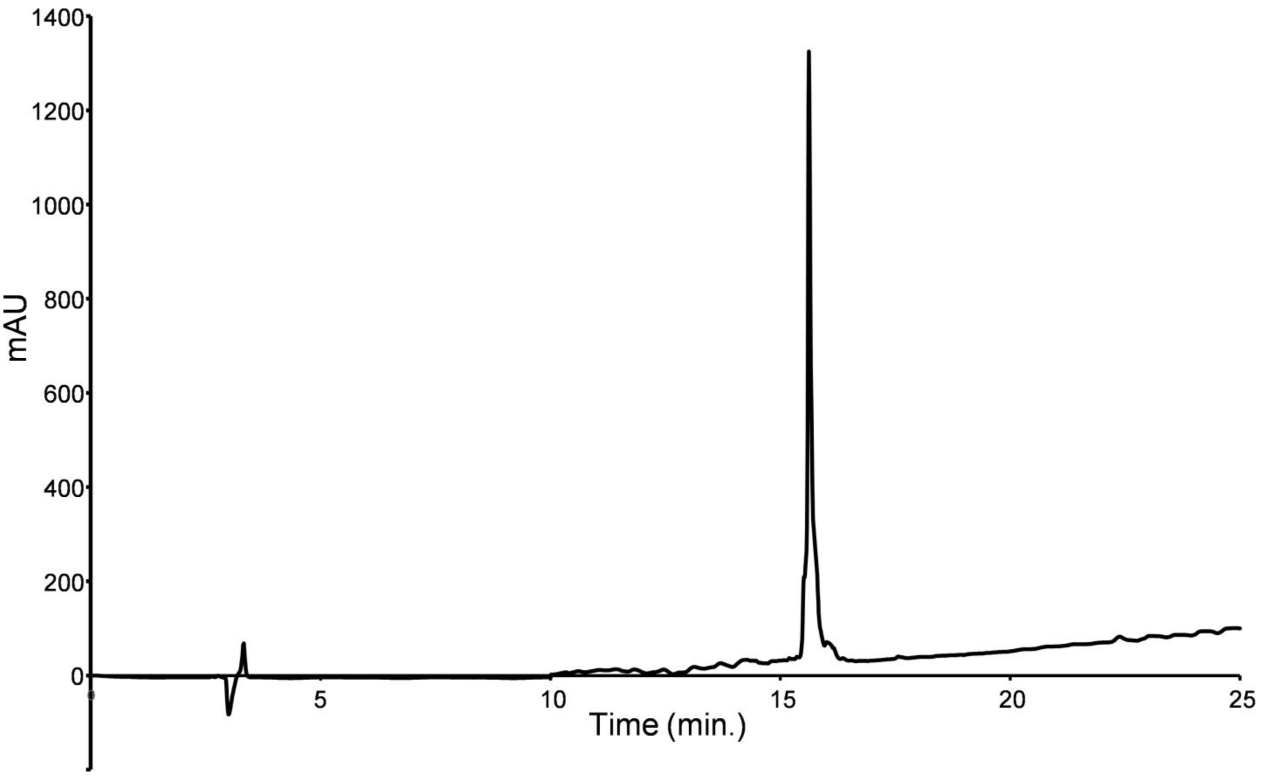


**Figure S1.** Chromatographic profile of $A\beta_{8\text{-}20}$ recorded at λmax=220 nm using a Phenomenex Kinetex XB- C18 analytical column (pore size: 100 Å, particle size: 5 µm, column length: 250 mm, internal diameter: 4,60 mm).

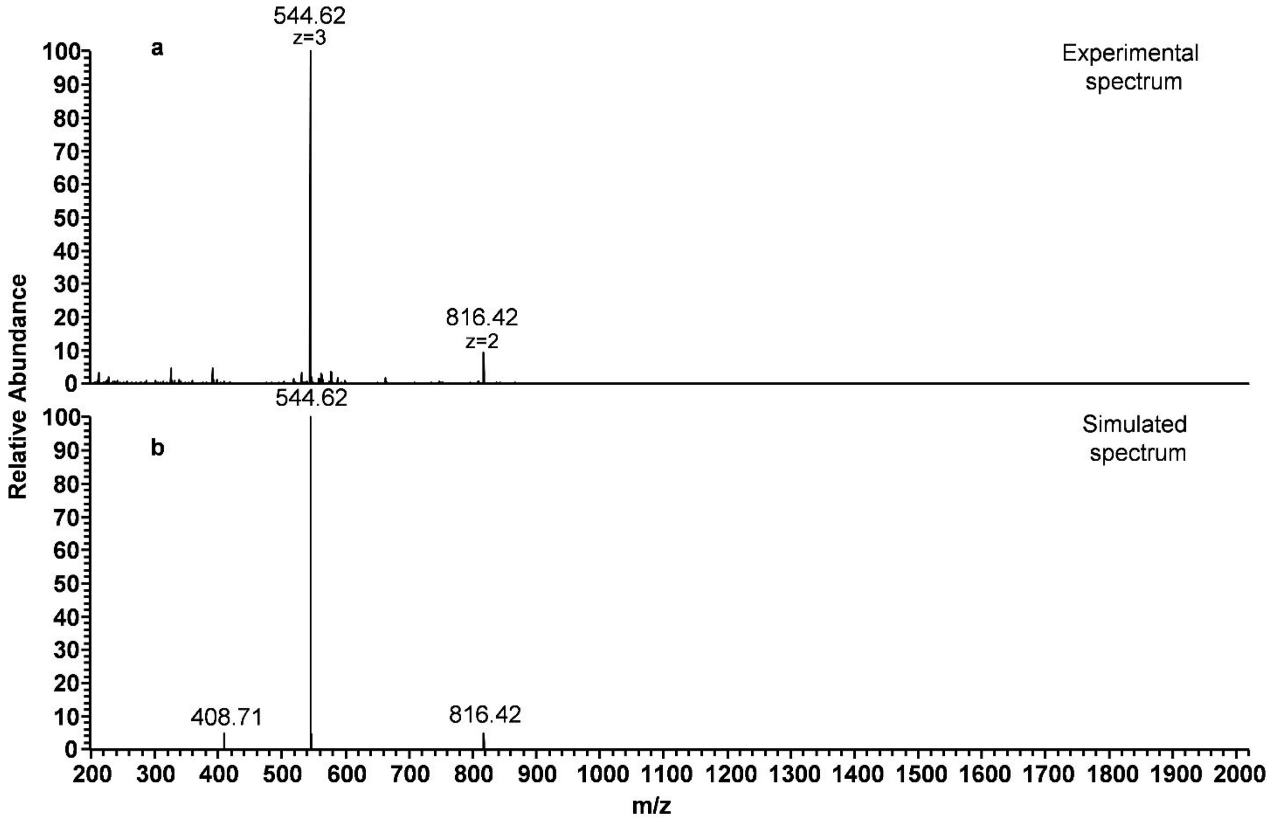


**Figure S2.** Panel a) FT-MS spectra (m/z range: 200–2000) of the Aβ$_{8-20}$ ($C_{peptide}$=5·10$^{-6}$M) at pH 7.0. Panel b) simulated m/z signals calculated by the molecular formula $C_{78}H_{110}N_{20}O_{19}$ corresponding to the Aβ$_{8-20}$ peptide.

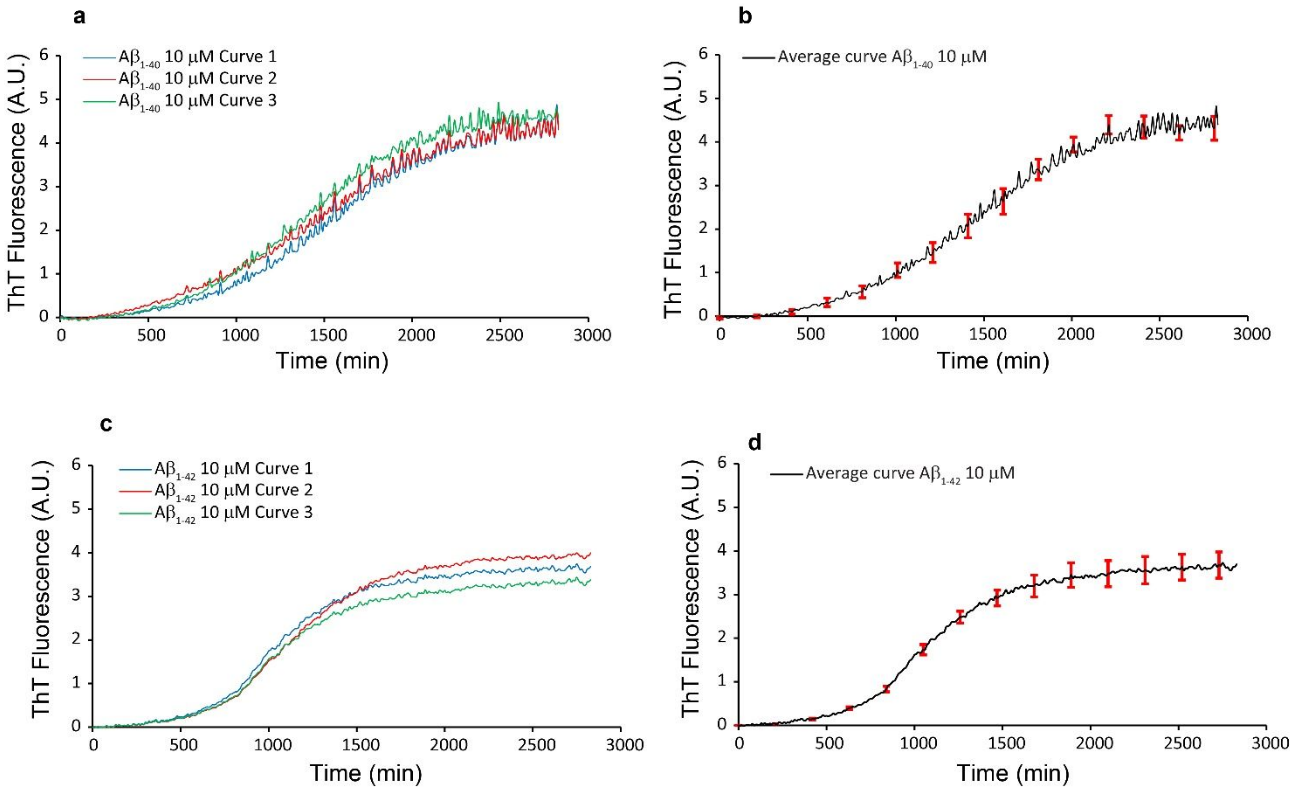


**Figure S3.** Panel a) three replicas of ThT measurement of $A\beta_{1-40}$ 10 μM. Panel b) average curve of $A\beta_{1-40}$ 10 μM with standard deviation. Panel c) three replicas of ThT measurement of $A\beta_{1-42}$ 10 μM. Panel d) average curve of $A\beta_{1-42}$ 10 μM with standard deviation.
